# A Map/INS/Wi-Fi Integrated System for Indoor Location-Based Service Applications

**DOI:** 10.3390/s17061272

**Published:** 2017-06-02

**Authors:** Chunyang Yu, Haiyu Lan, Fuqiang Gu, Fei Yu, Naser El-Sheimy

**Affiliations:** 1 College of Automation, Harbin Engineering University, Harbin 150001, China; yufei@hrbeu.edu.cn; 2 Department of Geomatics, University of Calgary, Calgary, AB T2N 1N4, Canada; hlan@ucalgary.ca (H.L.); elsheimy@ucalgary.ca (N.E.-S.); 3 Infrastructure Engineering, University of Melbourne, Melbourne, VIC 3010, Australia; fuqiangg@student.unimelb.edu.au

**Keywords:** non-holonomic constraints, map matching, map aiding, auxiliary value particle filter, indoor location based service system, cascade structure, non-holonomic constraints inertial navigation system (INS), Wi-Fi fingerprinting-aided navigation

## Abstract

In this research, a new Map/INS/Wi-Fi integrated system for indoor location-based service (LBS) applications based on a cascaded Particle/Kalman filter framework structure is proposed. Two-dimension indoor map information, together with measurements from an inertial measurement unit (IMU) and Received Signal Strength Indicator (RSSI) value, are integrated for estimating positioning information. The main challenge of this research is how to make effective use of various measurements that complement each other in order to obtain an accurate, continuous, and low-cost position solution without increasing the computational burden of the system. Therefore, to eliminate the cumulative drift caused by low-cost IMU sensor errors, the ubiquitous Wi-Fi signal and non-holonomic constraints are rationally used to correct the IMU-derived navigation solution through the extended Kalman Filter (EKF). Moreover, the map-aiding method and map-matching method are innovatively combined to constrain the primary Wi-Fi/IMU-derived position through an Auxiliary Value Particle Filter (AVPF). Different sources of information are incorporated through a cascaded structure EKF/AVPF filter algorithm. Indoor tests show that the proposed method can effectively reduce the accumulation of positioning errors of a stand-alone Inertial Navigation System (INS), and provide a stable, continuous and reliable indoor location service.

## 1. Introduction

Location-based services (LBS), which are accessible from mobile devices, have become increasingly important in recent years. LBS are widely used in a variety of contexts to provide services such as identifying the user’s location and performing mobile commerce for customers [1,2,3]. Currently, the common technologies for LBS can be divided into Radio Frequency (RF)-based and non-RF-based positioning methods [4]. The RF-based positioning methods include Wi-Fi-based positioning, cellular-based positioning, Bluetooth-based positioning, etc. On the other hand, the non-RF-based positioning methods include signage and maps positioning, inertial navigation, and acoustic positioning [5].

For commercial LBS, cost and convenience are the primary issues to be considered [6]. In this research, the non-RF-based positioning method, map, and INS positioning technologies are selected among various location method options. When compared with the RF-based positioning method, the non-RF-based positioning methods have the advantage of being low-cost and self-contained. Moreover, they do not require effort to install and maintain infrastructure, which must be considered carefully during the design and implementation of commercially used LBS systems [7]. Low-cost micro-electromechanical system (MEMS) sensors, which can provide a short-term accurate navigation solution without relying on any other sensors or instruments, are already built into smartphones and consumer devices [8,9,10]. However, the errors of the position derived from MEMS sensors grows very quickly with time, because of the integration processes. Therefore, aiding sensors or information are needed to provide an accurate, continuous, and stable navigation solution for indoor navigation service.

One of the most widely available approaches for pedestrian navigation are maps, because they do not require any additional infrastructure. Digital maps can be easily downloaded through scanning Quick Response (QR) codes or internet accessed smart-devices. The traditional way to use map for LBS is to plot the final solution in the present layer. However, map information can also be used in the computational process through Map Matching (MM) method and Map Aiding (MA) method [11,12,13]. MM method directly projects the estimated position coordinates to a previous know digital trajectory. The point to point, point to curve, and curve to curve methods can be used to match the estimated position to the nearest point. Different from MM, MA does not directly used to project the pedestrian’s position; however, MA utilizes map information to constrain the estimated solution through wall cross method. Both MM and MA have their benefits. Therefore, in this research, map aiding and map matching methods are innovatively combined to estimate a more accurate navigation solution.

For the LBS system, indoor map information is used as a boundary to constrain the INS/Map estimated solution. However, in large areas, such as airports and museums, there is not enough map information to constrain the INS derived position. Furthermore, considering the increasing coverage of free wireless Wi-Fi in public areas [14], Wi-Fi based positioning, which only uses pre-existing Wi-Fi infrastructures, may be used as additional aiding technology for indoor navigation. The main challenge for commercial LBS systems is to minimize the costs and use all the measurements/information effectively to provide a real-time, continuous, reliable positioning solution. Hence, a cascaded EKF/AVPF structure algorithm is designed in this research to optimally integrate the different sensors/information characteristics.

The organization of this paper is as follows: in Section 2, the methodology used in this research is presented. First, the model of INS navigation with non-holonomic constraints (NHC) motion constraints is given. Then, Wi-Fi fingerprinting method is introduced, and the Wi-Fi position is integrated with the INS/NHC estimated position. Third, a brief description of the cascade structure algorithm is provided, including lower layer EKF filter and upper layer AVPF filter, followed by application of the MM and MA methods on the upper filter. In Section 3, the performance of the proposed new approach is analyzed through field experiments. In Section 4, conclusions and future work are given. The acronyms used along this work can be found right after Section 4.

## 2. Methodology

In this research, we take advantage of the ubiquitous Wi-Fi, smartphone built-in IMU and the indoor map information to provide a low-cost and self-contained indoor navigation method. A two-layer KF/APF structure algorithm is designed and used to integrate the above information source. Moreover, a novel map matching and map aiding methods are combined to further improve the final solution by making full use of the indoor map information. In this methodology section, we explain how we used the Wi-Fi data and the IMU data to obtain the estimated positions. Then, we present how we combined/integrated these estimated positions together with the indoor map information.

**Figure 1:**
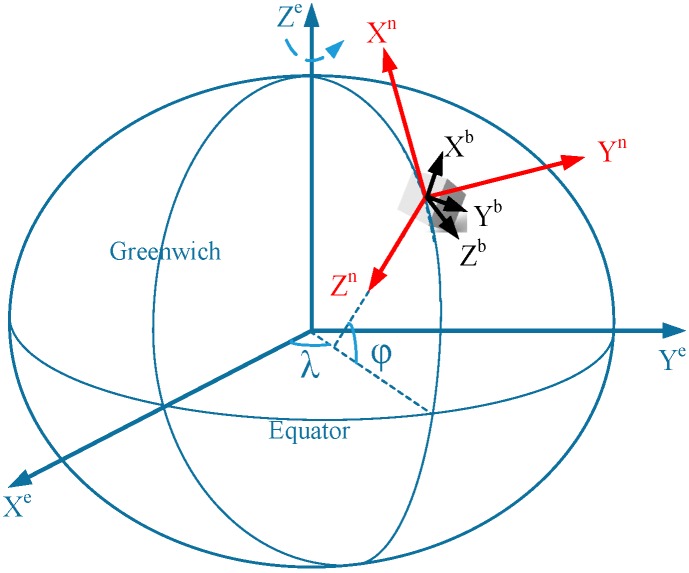
Frame definition used in the LBS.

Firstly, the coordinate systems which are used in this paper are defined as follows, and can be illustrated by Figure 1:

(1) The b-frame $\left(X_{b},Y_{b},Z_{b}\right)$ is the IMU (smartphone) body frame. Here, the body frame is positioned with its *y*-axis pointing forward (along the walking direction), *z*-axis pointing up and *x*-axis completing a right-handed orthogonal triad.
(2) The n-frame $\left(X_{n},Y_{n},Z_{n}\right)$ is the navigation frame. In this research, the NED frame is selected as the system navigation frame (n-frame). The *x*-axis (N) points towards the ellipsoidal North, and the *z*-axis is orthogonal to the reference ellipsoid and points downwards (D). The *y*-axis completes the right-handed orthogonal frame, thus pointing towards East (E).

Next in this section, we present the various techniques we used to deal with different kinds of information, as well as the two-layer structure we designed to combine the different kinds of information.

### 2.1. INS Navigation Solution

**Figure 2:**
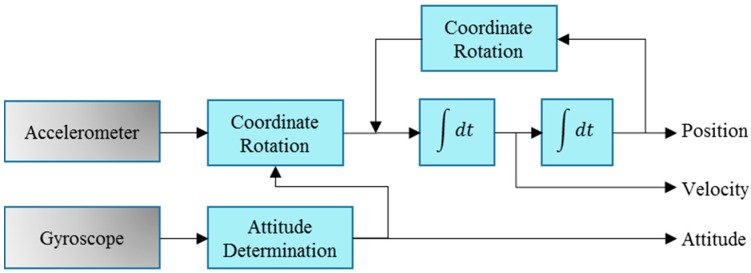
Flow diagrams for a strap-down inertial navigation system.

The INS navigation solution provides the primary information for the LBS; the Wi-Fi position and map information are then applied to aid the system on the basis of the INS solution. INS is typically implemented through the mechanization method. The mechanization equations are used in deriving the navigation information from the IMU measurements (i.e., specific force and angular rates). Figure 2 shows the schematic diagram of the INS mechanization method [9].

The motion of the moving object in the navigation frame is given as follows:

(1) $$\left[\begin{array}{c} {\dot{r}}^{n}\\ {\dot{v}}^{n}\\ {\dot{T}}_{b}^{n} \end{array}\right]=\left[\begin{array}{c} D^{-1}\;v^{n}\\ T_{b}^{n}\;f^{b}-2\;(\Omega_{ie}^{n}+\Omega_{en}^{n})+g^{n}\\ T_{b}^{n}\;(\Omega_{ib}^{b}-\Omega_{in}^{b}) \end{array}\right]$$

where $r^{n}={\left[\begin{array}{ccc} r^{x} & r^{y} & r^{z} \end{array}\right]}^{T}$ represents the INS derived position, $v^{n}={\left[\begin{array}{ccc} v^{x} & v^{y} & v^{z} \end{array}\right]}^{T}$ denotes the velocity, and $T_{b}^{n}$ is the rotation matrix describing the rotation of the body frame b relative to the n-frame. The rotation matrix can be used to derive the attitude vector (i.e., pitch, roll and heading). $f^{b}$ denotes the accelerometer measurements, and $\Omega^{b}$ is the skew symmetric matrix of the angular velocity $\omega^{b}$, which is measured by the gyroscope [15].

The error of the INS grows with time; therefore, NHC, a commonly used auxiliary information source, can be used to reduce the INS accumulated error [16,17]. NHC assumes that a moving platform cannot skid or jump, and the lateral and vertical speeds can be assumed to be zero. The forward, lateral, and vertical speeds update velocity for the INS in order to limit the INS velocity errors through EKF. The following equations are: (1) the EKF system update equation and (2) the measurement update equation:

(2) $$\begin{array}{c} \delta x_{k+1}^{INS}=\Phi_{k+1,k}^{INS}\delta x_{k}^{INS}+G_{k}^{INS}\omega_{k}^{INS}\\ \delta z_{k}^{INS/NHC}=H_{k}^{INS/NHC}\delta x_{k}^{INS}+v_{k}^{INS/NHC} \end{array}$$

Based on the mechanization Equation (1), we can obtain the linearized discrete INS error model [15]. The subscript *k* represents the update time *k*. The vector $\delta x_{k}^{INS}={\left[\begin{array}{ccc} \delta r^{n} & \delta v^{n} & \delta \varepsilon^{n} \end{array}\right]}^{T}$ is the INS error state including the error states of position, velocity, and attitude. $\omega_{k}^{INS}$ is the sensor noise vector, and $G^{ins}$ is the noise distribution matrix. $\Phi_{k+1,k}^{INS}$ is the discrete-time error state transition matrix from epoch k to k+1. $\delta z_{k}^{INS/NHC}$ is the measurement vector, which is the true velocity/position (NHC velocity) of the system minus the velocity vector derived from INS during the NHC update. $v_{k}^{INS/NHC}$ is the measurement noise matrix.

However, the NHC-aided INS-alone system has limited observation information. Therefore, before we designed an integrated navigation algorithm, it was necessary to analyze the system observability. Reference [18] deduces and discusses the observability of the NHC-INS system in detail. We know that the system observability of the NHC-aided INS system, specifically the system heading, is weak. Therefore, the ubiquitous Wi-Fi signal and map information are used to integrate with the INS solution.

### 2.2. System Initialization of INS

**Figure 3:**
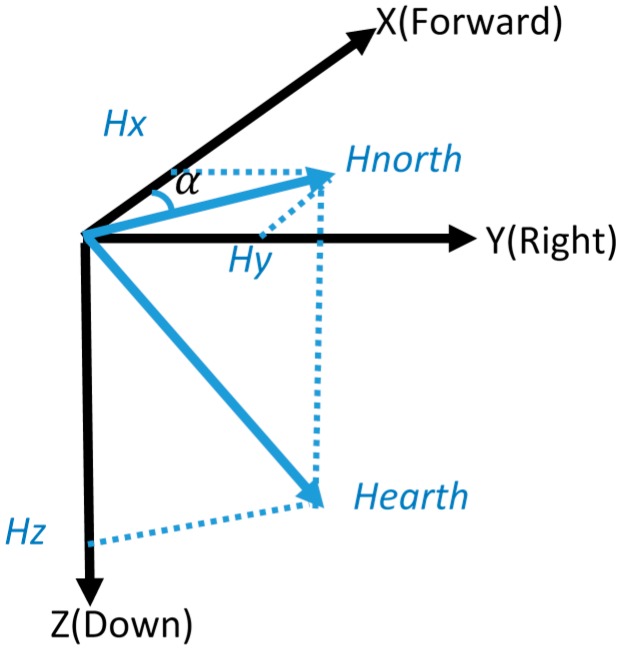
Heading estimation of magnetometer.

We know that the INS provides the relative position, which means that the initial position and attitude information are needed. There are two initialization modes for our system: (1) “manual mode”, which means the user can manually enter the initial position and heading according to the given map information, and (2) “program mode” in which the initial position is provided by the Wi-Fi solution, and assumes that the Wi-Fi signal is available. Moreover, the initial heading in this LBS is given by magnetometer, as shown in Figure 3. Then, the initial azimuth Ψ is given by:

(3) $$\Psi =\arctan\left(H_{y}/H_{x}\right)\mp D$$

where D is the magnetic declination, denoting the angle between the geographic north and the magnetic north, $H=\left(\begin{array}{ccc} H_{x} & H_{y} & H_{z} \end{array}\right)$ is the magnetometer-measured magnetic field vector composed of the three field components along the b-frame.

### 2.3. Wi-Fi Derived Solution

**Figure 4:**
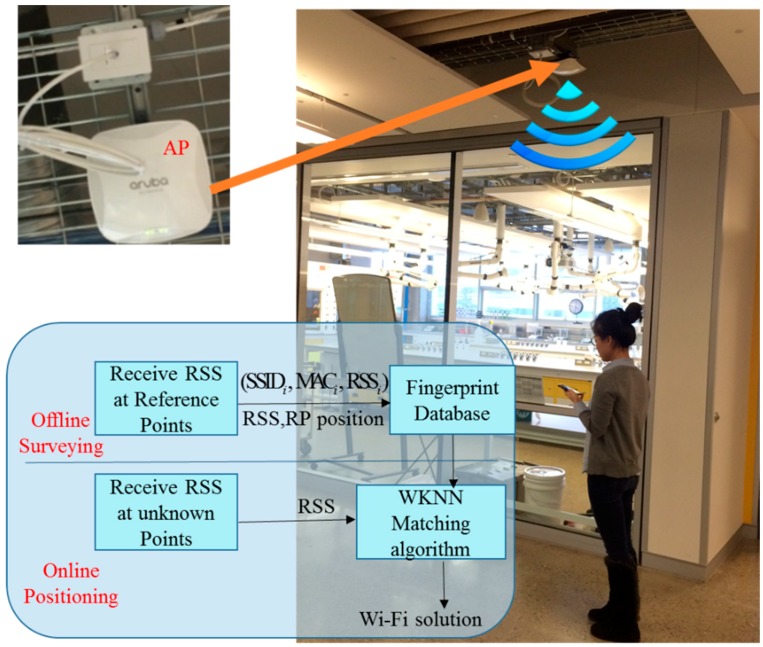
Fingerprinting-based Wi-Fi positioning Algorithm.

Fingerprinting and trilateration are two popular Wi-Fi based positioning methods, each of which has its own advantages and disadvantages. In this research, the fingerprinting method is used because it provides the users’ position without any knowledge of the access point’s (AP) location or signal-propagation model. As shown in Figure 4, fingerprinting-based Wi-Fi positioning usually has two operational steps: (1) the offline pre-survey, and (2) the online positioning [19].

In the first step, the Received Signal Strength values from the available access points and their observed position information are collected as fingerprints to populate a database [20]. Each fingerprint in the radio-map database is recorded as a vector, which normally has the following form: $S_{i}=\left\{\left({\mathrm{SSID}}_{1},{\mathrm{MAC}}_{1},{\mathrm{RSS}}_{1}),\ldots ,({\mathrm{SSID}}_{n},{\mathrm{MAC}}_{n},{\mathrm{RSS}}_{n}\right)\right\}$, where the Service Set Identifier (SSID) and the Media Access Control (MAC), respectively, present the name and the MAC address of a specific AP.

In the second step, the device’s position is estimated by comparing the measured vector of the RSS with the fingerprints in the pre-built database, in order to estimate and determine the closest fingerprints [21]. The Weighted K-Nearest Neighbor (WKNN) algorithm is used in estimating the optimal match between the device’s newly-collected RSSs and those RSSs stored in the radio-map database. Compared with traditional methods, such as the Nearest Neighbor (NN) algorithm and the K-Nearest Neighbor (KNN) algorithm, the weighted KNN (WKNN) takes more than one (*k*) neighbor into consideration and arranges the weight according to the Euclidean distances. The device’s current estimated position is updated by weighting the corresponding *k* reference coordinates (fingerprints) in the radio-map database using the following equation:

(4) $$\hat{p}=\sum_{j=1}^{k}\left(\frac{1/d_{j}}{\sum_{j=1}^{k}\left(1/d_{j}\right)}p_{j}\right)$$

where $\hat{p}$ is the estimated navigation coordinate; $p_{j}\;\left(j=1,2,\ldots ,k\right)$ is the position of the *j*-th selected reference coordinates in the database. $d=\left[d_{1}\;\ldots \;d_{m}\right]$, in which $d_{i}$ is the two-dimensional Euclidean distance between $\alpha_{i}$ and β, $d_{i}$ is defined as $d_{i}=\left|\alpha_{i}-\beta \right|$, (i=1,2,…,m). Vector $\alpha =\left\{\begin{array}{ccc} \alpha_{1} & \ldots & \alpha_{m} \end{array}\right\}$ represents the RSS set of the fingerprints’ database. The measured RSS values from *n* APs at an unknown location are expressed as a set $\beta =\left\{\begin{array}{ccc} \beta_{1} & \ldots & \beta_{m} \end{array}\right\}$, *n* is the number of the access point. *K* is the setting number of the K-nearest neighbors (points) with the smallest distances to β.

### 2.4. Wi-Fi/INS/NHC Kalman Filter

When the Wi-Fi position solution is available, the measurement update equation for the EKF in Equation (2) will be updated as:

(5) $$\dot{\delta z_{k}^{INS/NHC/Wi-Fi}}=H_{k}^{INS/NHC/Wi-Fi}\delta x_{k}^{INS}+v_{k}^{INS/NHC/Wi-Fi}$$

where $\delta z_{k}^{INS/NHC/Wi-Fi}$ is the measurement vector, which is the true velocity/position (NHC velocity/Wi-Fi position) of the system minus the velocity/position vector derived from INS during the NHC/Wi-Fi update. $v_{k}^{INS/NHC/Wi-Fi}$ is the measurement noise matrix. The measurement matrix is $H_{k}^{INS/NHC/Wi-Fi}=\left[\begin{array}{ccc} I_{3\times 3} & I_{3\times 3} & 0_{3\times 3} \end{array}\right]$. Everytime the state error $\delta x_{k}^{INS}$ is updated, the errors are applied to the INS navigation solution; then, $\delta x_{k}^{INS}$ is reset, which means the error state prediction procedure is no longer required. The complementary characteristics of MEMS sensors and Wi-Fi enable an efficient integration for indoor navigation applications. Thus, if a Wi-Fi signal is available, we estimate a more accurate INS solution from the lower filter. Subsequently, the number of particles used in the upper filter decreases, which can indirectly increase the computational speed of the system. However, if no Wi-Fi signal is available, an indoor map is still the main auxiliary information of the system.

### 2.5. Two-Layer KF/PF Structure

**Figure 5:**
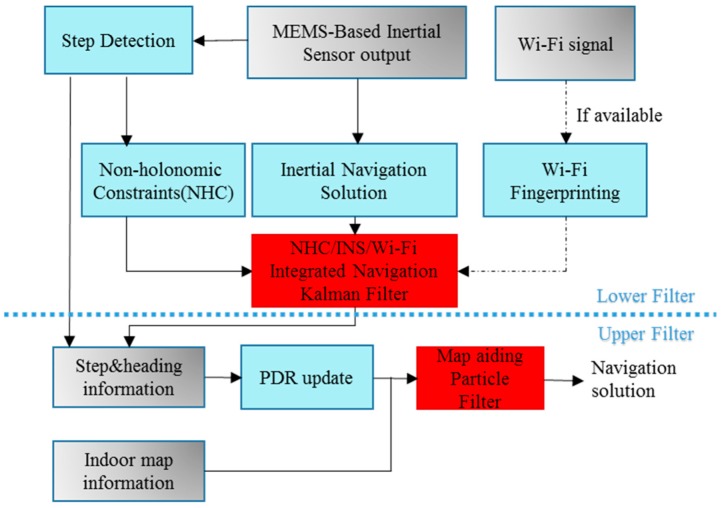
Map/INS/Wi-Fi integration algorithm using KF and PF.

This research proposes a two-layer KF/PF structure algorithm as shown in Figure 5. On the upper layer of the algorithm, a PF is used to introduce the map information to the primary Wi-Fi/INS integrated solution. The PF is easy and convenient for adding additional map information, and can accommodate arbitrary sensor characteristics, motion dynamics, and noise distributions. Moreover, the number of samples used in PF can be controlled to suit the available computational resources in some enhanced versions of PF. The step and heading information calculated from the primary Wi-Fi/INS solution are utilized to perform the Pedestrian Dead Reckoning (PDR) update; the update rate is the same as the low step detection rate. After this step, the indoor map information is used as new measurement to re-set the weights of particles and obtain a map-constrained navigation solution.

To take full advantage of indoor map information, the map-matching method is also used to project the INS/Map integrated solution to the map. However, the PF algorithm also decreases the computational efficiency, and may face particle failure problems. To overcome these implementation issues of PF, the KF is used on the bottom layer of the algorithm, NHC and Wi-Fi estimated position (if Wi-Fi is available) are used as inputs to the KF to correct the preliminary INS navigation solution. Compared with the other traditional particle filters, PF with a two-layer KF/PF structure has low computational burden, because the update rate of PF has been changed from “IMU data output rate 50 Hz” to “step detection update rate”.

### 2.6. Map Aiding through Auxiliary Value PF

Indoor map information is used to constrain the primary INS solution through the PF, which is numerically implementation of Bayesian estimator. A set of particle samples are used to represent the posterior density $p(x_{t}|Y_{t})$ through the Monte Carlo approach [22,23]:

(6) $$p(x_{t}|Y_{t})\approx \overset{N}{\underset{i=1}{\sum }}w_{t}^{\left(i\right)}\delta \left(x-x_{t}^{\left(i\right)}\right)$$

where *t* is the time index, $x_{t}$ is the state, $Y_{t}$ is the measurement set. $w_{t}^{\left(i\right)}$ is the *i*-th particle’s weight at time *t*, and can be represented as:

(7) $$w_{t}^{\left(i\right)}\propto w_{t-1}^{\left(i\right)}\frac{p(Y_{t}|x_{t}^{\left(i\right)})p(x_{t}^{\left(i\right)}|x_{t-1}^{\left(i\right)})}{q(x_{t}^{\left(i\right)}|x_{t-1}^{\left(i\right)},y_{t})}$$

where *q* is the importance density function. Traditional PF methods select the state transition probability density function as the importance density function of PF, and the traditional PF methods do not take the measurements into consideration, and therefore may cause unsatisfactory sampling results. This can result in many particle weights at values of either zero or close to zero, and few particles are duplicated during the resampling processes. Therefore, the PF method loses diversity, and suffers from particle impoverishment, as previously reported in [24].

In this research, Auxiliary Value Sequential Importance Resampling (ASIR) PF is applied instead of the traditional SIR PF. Different from the state transition probability density function, AVPF takes the measurements into consideration and provides an efficient method for solving the particle impoverishment problem [25,26]. On the other hand, particles that do not match the current measurements are pre-tested and deleted. PF consists of three phases: system propagation, measurement update, and resampling (when required). The posterior probability density function of $x_{k}$ can be represented as:

(8) $$p\left(x_{k}|z_{0:k}\right)\propto p\left(z_{k}|x_{k}\right)\cdot p\left(x_{k}|z_{0:k-1}\right)=\overset{N_{s}}{\underset{i=1}{\sum }}\left[w_{k-1}^{i}p\left(z_{k}|x_{k}\right)\right]p\left(x_{k}|x_{k-1}^{i}\right)$$

Introducing the auxiliary variable ζ to represent the component index in the above equation:

(9) $$p\left(x_{k},\varsigma_{k-1}^{i}|z_{0:k}\right)\propto p\left(z_{k}|x_{k}\right)\cdot p\left(x_{k},\varsigma_{k-1}^{i}|z_{0:k-1}\right)=\overset{N_{s}}{\underset{i=1}{\sum }}\left[w_{k-1}^{i}p\left(z_{k}|x_{k}\right)\right]p\left(x_{k}|x_{k-1}^{i}\right)$$

then we can select the *importance density function:*

(10) $$q\left(x_{k},\varsigma_{k-1}^{i}|z_{0:k}\right)=q\left(\varsigma_{k-1}^{i}|z_{0:k}\right)\;q\left(x_{k},\varsigma_{k-1}^{i}|z_{0:k-1}\right)\propto \overset{N_{s}}{\underset{i=1}{\sum }}\left[w_{k-1}^{i}p\left(z_{k}|u_{k|k-1}^{i}\right)\right]p\left(x_{k}|x_{k-1}^{i}\right)$$

in which, $\left(z_{k}|u_{k|k-1}^{i}\right)$ is the statistic of $x_{k}$ given $x_{k-1}^{i}$, and it is related with $p\left(x_{k}|x_{k-1}^{i}\right)$. Usually, $u_{k|k-1}^{i}$ can be set as the expectation of $p\left(x_{k}|x_{k-1}^{i}\right)$. From the importance resampling method, we can obtain a set of new particles ${\left\{\left(x_{k}^{i},\varsigma_{k-1}^{i}\right),w_{k}^{i}\right\}}_{i=1}^{N_{s}}∼q\left(x_{k},\varsigma_{k-1}^{i}|z_{0:k}\right)$. Ignoring the auxiliary variable ζ, we can obtain the particle set ${\left\{\left(x_{k}^{i},w_{k}^{i}\right)\right\}}_{i=1}^{N_{s}}$, in which $w_{k}^{i}$ is the weight of the particles:

(11) $$w_{k}^{i}\propto \frac{p\left(x_{k}^{i},\varsigma_{k-1}^{i}=i|z_{0:k}\right)}{q\left(x_{k}^{i},\varsigma_{k-1}^{i}|z_{0:k}\right)}=\frac{p\left(z_{k}|x_{k}^{i}\right)}{p\left(z_{k}|u_{k|k-1}^{i}\right)}$$

Therefore, the process of AVPF can be summarized as:

(1) Calculating the auxiliary variable $u_{k|k-1}^{i}$ for each particle.
(2) Calculating the auxiliary weight of each particle: ${\hat{w}}_{k-1}^{i}\propto w_{k-1}^{i}p\left(z_{k}|u_{k|k-1}^{i}\right)$.
(3) Normalizing the weight: $\tilde{w_{k}^{i}}={\hat{w}}_{k-1}^{i}/\overset{N_{s}}{\underset{i=1}{\sum }}{\hat{w}}_{k-1}^{i}$.
(4) Resampling according to the normalized auxiliary weight $\tilde{w_{k}^{i}}$, but only the sequence number $\varsigma_{k-1}^{i}$ is needed.
(5) Importance sampling (Prediction):$\;x_{k}^{i}∼q\left(x_{k},\varsigma_{k-1}^{i}|z_{0:k}\right)=p(x_{k}^{i}|x_{k-1}^{\varsigma_{k-1}^{i}})$.
(6) Calculating the weight: $w_{k}^{i}\propto \frac{p\left(x_{k}^{i},\varsigma_{k-1}^{i}=i|z_{0:k}\right)}{q\left(x_{k}^{i},\varsigma_{k-1}^{i}|z_{0:k}\right)}=\frac{p\left(z_{k}|x_{k}^{i}\right)}{p\left(z_{k}|u_{k|k-1}^{i}\right)}$ and normalizing the value to obtain both the particles and their weight ${\left\{\left(x_{k}^{i},\tilde{w_{k}^{i}}\right)\right\}}_{i=1}^{N_{s}}$.

The Dead Reckoning (DR) positioning technique is used to build the particle filter system update model. Essentially, the Pedestrian Dead Reckoning (PDR) method determines a new position by utilizing the knowledge of a previously known position, together with the current travelled distance and heading information [27,28]. In this research, step detection of the upper filter is performed during the INS mechanization process in the lower filter, using the method in [29]. The stride length and heading estimation results derived from the lower KF are the measurements of the upper PF. Therefore, the system model for PF can be written as:

(12) $$\begin{array}{c} E_{k+1}^{i}=E_{k}^{i}+S_{k}^{i}\sin\left(\Psi_{k}^{i}\right)\\ N_{k+1}^{i}=N_{k}^{i}+S_{k}^{i}\cos\left(\Psi_{k}^{i}\right) \end{array}$$

in which $S_{k}^{i}$ is the stride length of the user, and $\Psi_{k}^{i}$ is the heading. The superscript *i* denotes the *i*-th particle of PF, and the subscript *k* denotes the *k*-th step of the PDR update. $E_{k+1}^{i}$ and $N_{k+1}^{i}$ are the East and North position of the system.

For the measurement update process, two-dimensional indoor architectural map information is used as a new measurement to update the weight of the particles $w_{k}^{\left(i\right)}$. A Cross-wall method based on indoor map aiding is used to update the particle weight [30]. Specifically, if the new particle has intersection with the wall after system propagation, then this particle is invalid, and assigned a zero weight (i.e., $w_{k}^{\left(i\right)}=0$). If a wall is not intersected by the particle during the propagation step, the new generated particle is valid, and the weight of the particle remains the same, as with the previous step.

### 2.7. Map Matching

The map-matching method is the last step of the navigation algorithm used to re-correct the estimation error after applying the PF-based map-aiding method. The predicted position from PF is projected to the digital map database. Depending on the format of the indoor digital map, different map matching algorithms can be used. In this paper, the point-to-point map-matching method is used because of the simple format requirements of the digital map. The algorithm projects the estimated location, $P\left(X_{P},Y_{P}\right)$, to the closest link in the network using the distance form Equation (13) [31]:

(13) $$X_{P}=\frac{\left[AX_{e}+BY_{e}]+B(X_{1}Y_{2}-X_{2}Y_{1}\right)}{[A^{2}+B^{2}]A^{-1}}$$

(14) $$Y_{P}=\frac{\left[AX_{e}+BY_{e}]+A(X_{1}Y_{2}-X_{2}Y_{1}\right)}{[A^{2}+B^{2}]B^{-1}}$$

in which $\left(X_{e},Y_{e}\right)$ is the position estimated from the MA-based APF algorithm, $\left(X_{1},Y_{2}\right)$ is the start-point, and $\left(X_{2},Y_{2}\right)$ is the end-point of the closest line segment in the digital map. Additionally, $A=\left(X_{2}-X_{1}\right)$, and$\;B=\left(Y_{2}-Y_{1}\right)$. We obtain the closest line segment using Equation (15):

(15) $$Distance=\frac{\left|-X_{e}B+Y_{e}A+(X_{1}Y_{2}-X_{2}Y_{1})\right|}{\sqrt{A^{2}+B^{2}}}$$

Therefore, the final estimated position solution is the set of these projection points: $P\left(X_{P},Y_{P}\right)$.

In summary, in this research, we present a Map/INS/Wi-Fi integrated indoor navigation method, and a two-layer KF/APF structure is proposed to combine multiple information sources. From the design of the system methodology, we know that this is a self-contained and low cost system. Moreover, the low PF update rate can decrease the system computational burden to a certain extent. In the following section, we test the accuracy of the proposed method.

## 3. Experiment and Analysis

**Figure 6:**
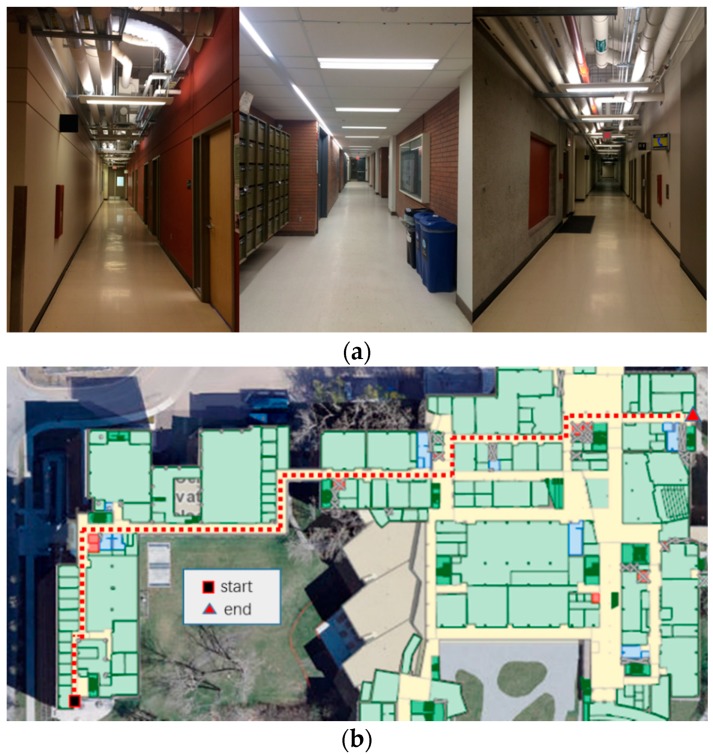
(**a**) Environment of the ENG Test; (**b**) The designed experiment trajectory on the first floor in ENG building.

**Figure 7:**
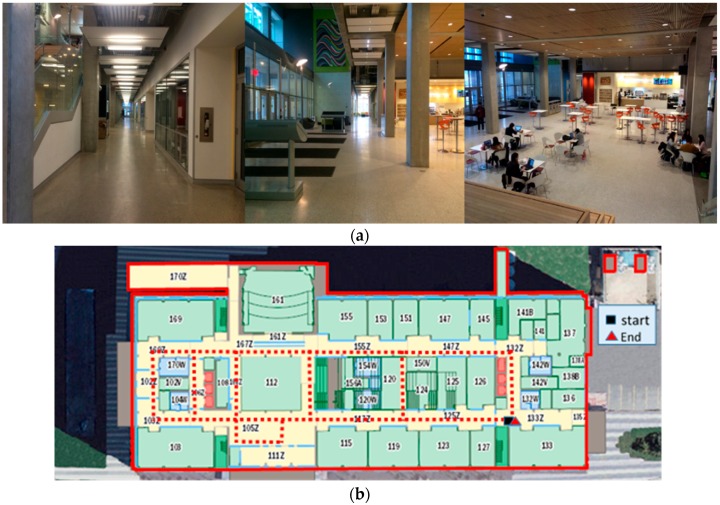
(**a**) Environment of the EEEL Experiment; (**b**) The designed experiment trajectory on the first floor in EEEL building.

The validity and feasibility of the proposed algorithm are confirmed through conducting indoor experiments in the basement floor of Engineering (ENG) building and the first floor of the Energy Environment Experiential Learning (EEEL) building at the University of Calgary. The time duration of these tests is approximately 30 min. Figure 6a and Figure 7a show the experiment environment for the tests in the ENG and EEEL building, respectively. Figure 6b and Figure 7b are the corresponding designed experiment trajectory plotted on the two-dimension digital map of the first floor.

From Figure 7a, we can see that there is a hall in EEEL building (112 in Figure 7b). For this area, not much architecture map information can be used to constrain the INS derived solution. Also, for deep indoor areas, there is no GNSS signal can be used to provide positional information.

The proposed algorithm has been implemented on the Samsung Galaxy Note 4 smartphone which includes the sensors listed in Table 1.

**Figure 8:**
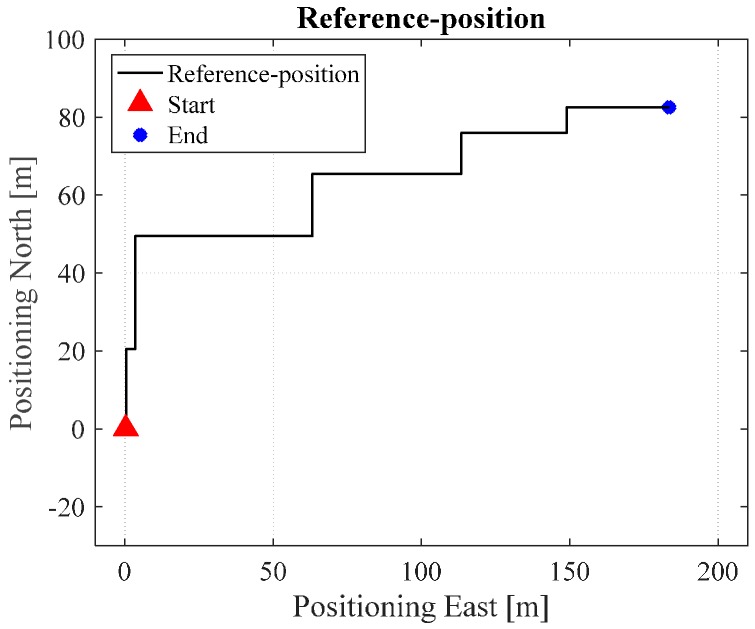
Reference trajectory in ENG.

**Figure 9:**
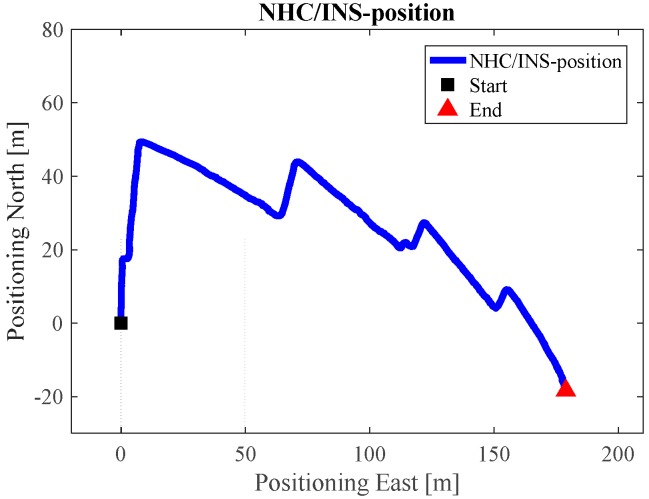
NHC/INS estimated solution in ENG.

**Figure 10:**
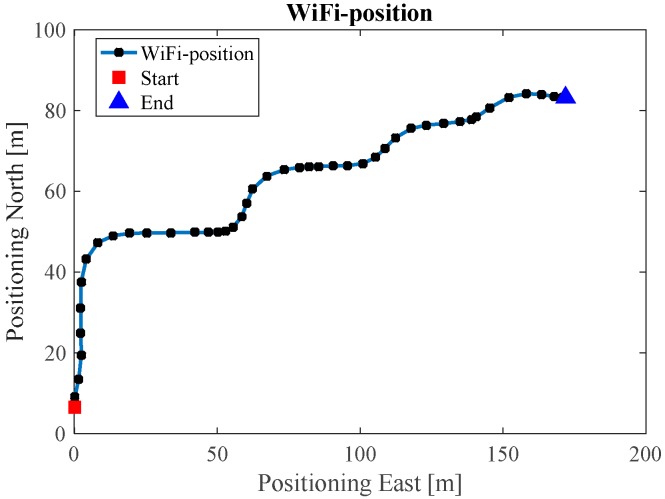
Wi-Fi estimated position in ENG.

**Figure 11:**
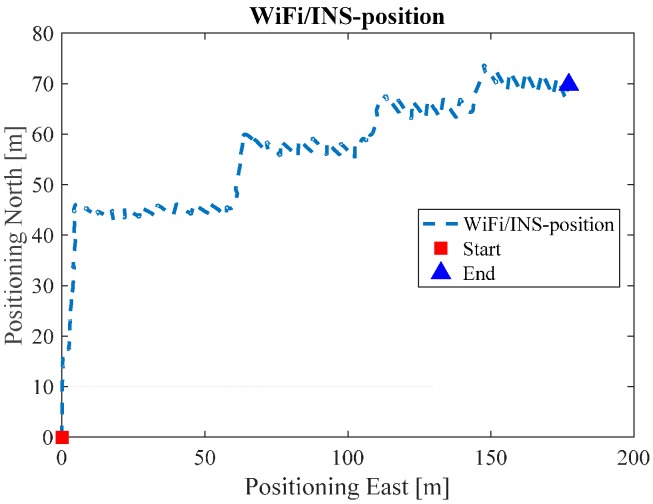
Wi-Fi/INS integrated position in ENG.

**Figure 12:**
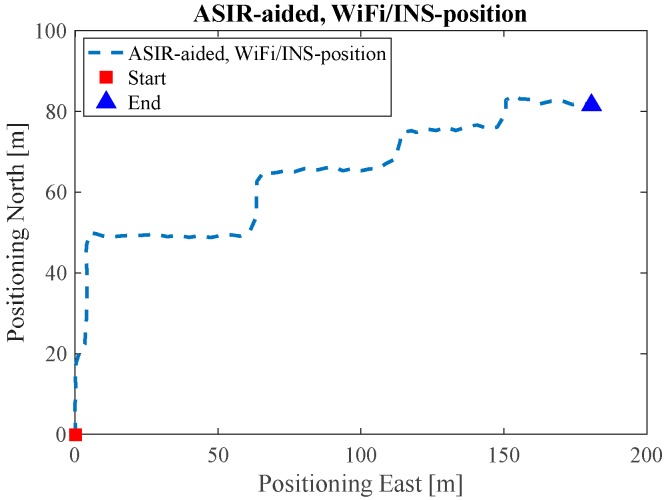
Map/INS/Wi-Fi solution in ENG.

**Figure 13:**
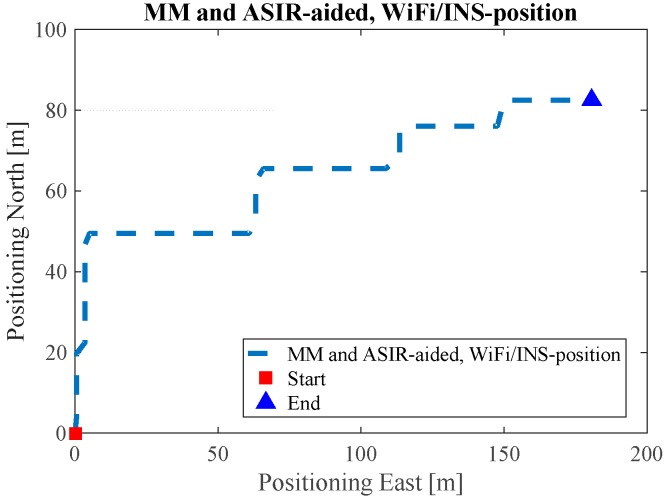
MM/Map/INS/Wi-Fi solution in ENG.

In order to quantitatively analyze the performance of the proposed algorithm, a “heading lock” PDR method is used in generating reference trajectories. In this mode, it is assumed that the user’s walking heading for the PDR update is “locked” to a specific value according to the indoor map. This mode then uses the specific corners and intersections as “landmarks” to further correct the PDR-derived positions. From this method, we obtain the reference trajectories of the tests in ENG and EEEL, illustrated in Figure 8. Figure 9, Figure 10, Figure 11, Figure 12 and Figure 13 illustrate the estimated solutions of test in ENG building from different kinds of methods.

Figure 9 is the INS/NHC derived position without auxiliary Wi-Fi position or map information for the test in the ENG building. If we compare Figure 7 with the reference trajectory in Figure 6, it is evident that INS-alone system cannot provide realistic estimated position solutions, even with the NHC velocity constraint. The reason for this is when the velocity is used to correct the system error, the heading of the system is unobservable, which is also confirmed by the observability analysis conducted in [32]. Therefore, aiding information is needed to further correct the error of the system.

Figure 10 illustrates the Wi-Fi fingerprint estimated positions, where each black dot represents one Wi-Fi estimated position. Figure 11 is the INS/Wi-Fi integrated positions.

Figure 12 shows the Map/Wi-Fi/INS integrated solution using the PF-based map-aiding method. Figure 13 shows the map-matching and map-aiding combined estimated solution. By comparing Figure 12 with the reference trajectory in Figure 8, the estimated trajectory closely matches the reference trajectory.

**Figure 14:**
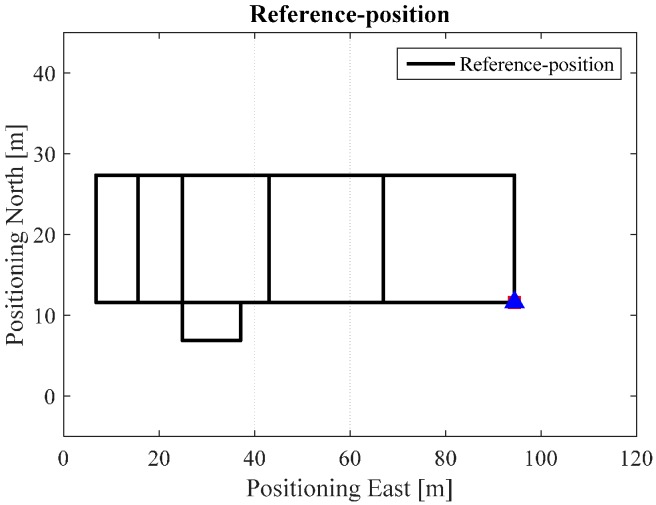
Reference trajectory of the EEEL test.

**Figure 15:**
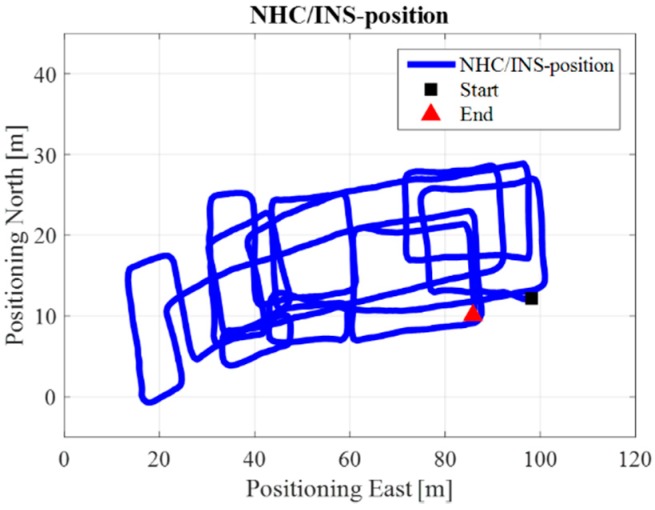
INS estimated solution in EEEL.

**Figure 16:**
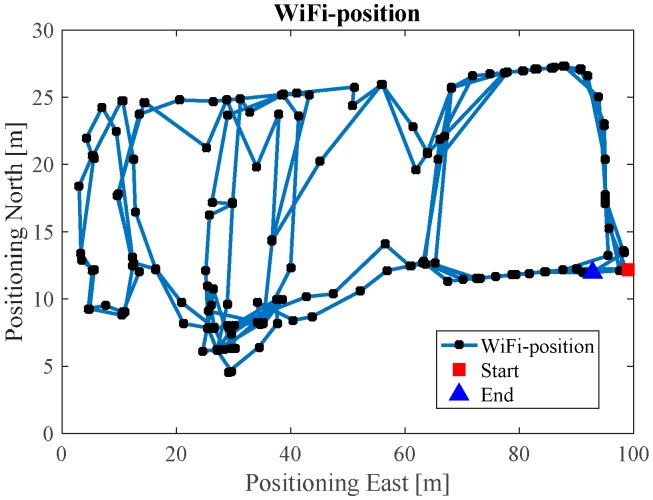
Wi-Fi estimated position in EEEL.

**Figure 17:**
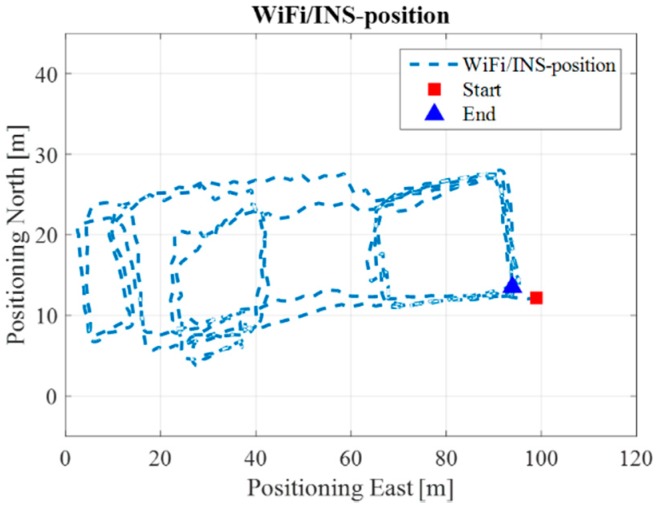
Wi-Fi/INS integrated position in EEEL.

**Figure 18:**
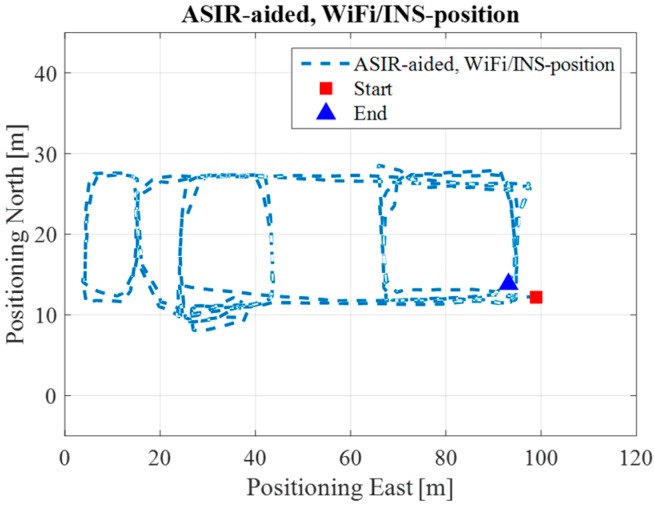
Map/INS/Wi-Fi solution in EEEL.

**Figure 19:**
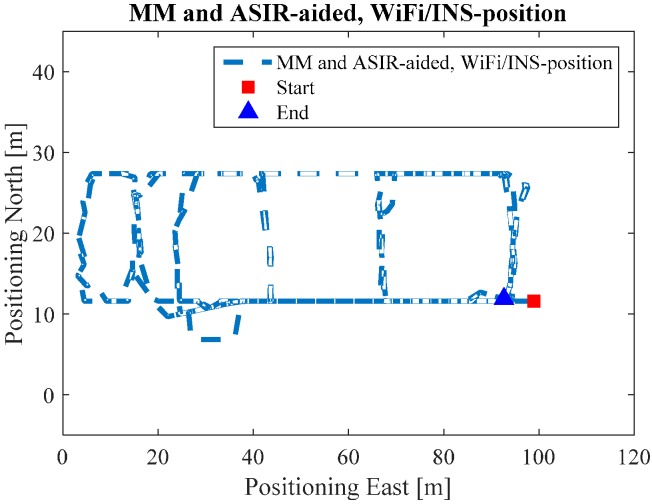
MM/Map/INS/Wi-Fi solution in EEEL.

For the test in the EEEL building, Figure 14 is the reference trajectory. Figure 15, Figure 16, Figure 17, Figure 18 and Figure 19 illustrate the estimated solutions from different kinds of methods. Figure 15 is the INS/NHC derived position without auxiliary Wi-Fi position or map information. Comparing Figure 15 with the reference trajectory in Figure 14, it is also evident that INS-alone system cannot not satisfy the user requirements.

Figure 16 illustrates the Wi-Fi fingerprint estimated positions, and Figure 17 is the INS/Wi-Fi integrated positions. From Figure 16, we can see that the Wi-Fi fingerprint solution contains positional errors that distort the whole trajectory. However, when we aid the Wi-Fi solution with the PDR, the results in Figure 17 shows that the integrated algorithm can complement each other, providing a better lower filter solution. However, the integrated Wi-Fi/PDR solution still contains positional drift in the trajectory.

Figure 18 shows the Map/Wi-Fi/INS integrated solution using the PF-based map-aiding method, and Figure 19 shows the map-matching and map-aiding combined estimated solution. By comparing Figure 18 with Figure 14, the estimated trajectory closely matches the reference trajectory. Additionally, when we combine the map-matching method with the map-aiding method, the user’s trajectory will further converge; when MM method is applied the RMS error improves by 1 m, respectively.

Finally, Root-Mean-Square (RMS) values are calculated to analyze the estimated ENG and EEEL test results using the four methods. The RMS error of the proposed methods are computed, in order to see the difference between the estimated navigation solutions using our methods and the reference true trajectory. The following equation is used to calculate the root square mean error:

(16) $$RMSD=\sqrt{\frac{\sum_{k=1}^{n}{\left(x_{1,k}-x_{2,k}\right)}^{2}}{n}}$$

where $x_{1,k}$ is the reference position, and $x_{2,k}$ is the estimated position from the proposed methods. Table 2 shows the RMS values of INS/NHC solution, Wi-Fi fingerprint estimated solution, INS/Wi-Fi integrated solution, Map/INS/Wi-Fi integrated solution through map-aiding method, and Map/INS/Wi-Fi integrated solution through the map-aiding and map-matching methods for both the ENG test and EEEL test. After integrating with the Wi-Fi estimated solution, the accuracy of the lower filter solution improves from 23.1 to 7.2, and 16.8 to 7.3. When indoor map information is added into the system, this accuracy further improved by more than 2 m. Additionally, when we add the map- matching method into the system, the RMS error of the system can be reduced to less than 5 m. Using the proposed method, we can obtain similar RMS value with [33,34,35], but have a low cost and self-contained system.

Another thing needs to be mentioned is that, thousands of particles or even more are needed for traditional PF to implement indoor navigation [36,37]; however, in this research, we need less particles than traditional PF. Using the proposed method in this research, for the test in EEEL building, 2000 particles are needed; for the test in ENG building, only 1000 particles are needed. The number of particles used in the research can indirectly demonstrate that the proposed method has low computational burden.

## 4. Conclusions

This paper presents a two-layer EKF/AVPF structure algorithm to integrate MEMS/Wi-Fi/Map integrated indoor LBS applications. The two-layer EKF/AVPF structure algorithm is used to increase the system efficiency and decrease the system computational burden. The two-layer structure can take advantage of both PF and EKF, ensuring the utilization rate of inertial sensor data, while reducing the update frequency of PF. Therefore, we conclude that the INS/Wi-Fi integrated algorithm can improve the accuracy of the lower filter INS solution. In addition, the MA-based AVPF method improved the accuracy of the INS-derived navigation solution. Furthermore, by adding the MM algorithm, the MA-based AVPF results were optimized. This research applies free ubiquitous Wi-Fi signal and indoor map information, in order to correct the INS accumulative errors. This can decrease the cost of LBS and has significant meaning for its popularization. Moreover, based on RMS analysis, the system accuracy has been effectively improved, and the RMS error was reduced within 5 m.

In future, we will test the algorithm in multiple floor-plan environments, so the user will have continuous navigation service, while moving between several floors. Then, all the proposed algorithms will be programed into a smartphone platform in real time. Moreover, we will design appropriate algorithms and perform real-time experiment to directly demonstrate that the proposed method has a low computational burden.

## Figures and Tables

**Table 1:** Sensors of the Samsung S4.

Sensor	Model
**Accelerometer**	STM K3DH
**Gyroscope**	STM K3G
**Compass**	AKM AK8975
**Barometer**	Bosh BMP182

**Table 2:** RMS values of 30 min test in EEEL building.

Methods	RMS-ENG	RMS-EEEL
**INS/NHC**	23.1	16.8
**Wi-Fi**	5.8	9.2
**INS/Wi-Fi**	7.2	7.4
**MA/INS/Wi-Fi**	4.7	5.7
**MM + MA/INS/Wi-Fi**	3.0	4.8

## Abbreviations

AVPF	Auxiliary Value Particle Filter
AP	Access Point
DR	Dead Reckoning
EEEL	Energy Environment Experiential Learning
ENG	Engineering
EKF	Extended Kalman Filter
IMU	Inertial Measurement Unit
INS	Inertial Navigation System
KNN	K-Nearest Neighbor
LBS	Location Based Services
MAC	Media Access Control
MEMS	Micro-Electromechanical System
NHC	Non-Holonomic Constraints
PDR	Pedestrian Dead Reckoning
QR	Quick Response
RMS	Root-Mean-Square
RF	Radio Frequency
RSSI	Received Signal Strength Indicator
SSID	Service Set Identifier
WKNN	Weighted K-Nearest Neighbor

## References

[1] Junglas I.A., Watson R.T. (2008). Location-based services. Commun. ACM.

[2] Rao B., Minakakis L. (2003). Evolution of mobile location-based services. Commun. ACM.

[3] Asghar M.Z., Ahmad S., Yasin M.R., Qasim M. (2014). A Review of Location Technologies for Wireless Mobile Location-Based Services. J. Am. Sci..

[4] Yassin M., Rachid E. A survey of positioning techniques and location based services in wireless networks. Proceedings of the 2015 IEEE International Conference on Signal Processing, Informatics, Communication and Energy Systems (SPICES).

[5] Karimi H.A. (2015). Indoor Wayfinding and Navigation.

[6] Niu B., Li Q., Zhu X., Cao G., Li H. Enhancing privacy through caching in location-based services. Proceedings of the 2015 IEEE Conference on Computer Communications (INFOCOM).

[7] Lorincz K., Welsh M. (2007). MoteTrack: A robust, decentralized approach to RF-based location tracking. Pers. Ubiquitous Comput..

[8] Yu C., Zhuang Y., El-Sheimy N., Li Y., Lan H. (2015). A novel kalman filter with state constraint approach for the integration of multiple pedestrian navigation systems. Micromachines.

[9] Shin E.-H., El-Sheimy N. Accuracy Improvement of Low Cost INS/GPS for Land Applications. Proceedings of the 2002 National Technical Meeting of The Institute of Navigation.

[10] Godha S. (2006). Performance Evaluation of Low Cost MEMS-Based IMU Integrated with GPS for Land Vehicle Navigation Application. Master’s Thesis.

[11] Stéphane B., Klepal M. Indoor PDR performance enhancement using minimal map information and particle filters. Proceedings of the 2008 IEEE/ION Position, Location and Navigation Symposium.

[12] Link J.A.B., Smith P., Viol N., Wehrle K. Footpath: Accurate map-based indoor navigation using smartphones. Proceedings of the 2011 International Conference on Indoor Positioning and Indoor Navigation (IPIN).

[13] Hashemi M., Karimi H.A. (2016). A weight-based map-matching algorithm for vehicle navigation in complex urban networks. J. Intell. Transp. Syst..

[14] Zanella A., Bui N., Castellani A., Vangelista L., Zorzi M. (2014). Internet of things for smart cities. IEEE Internet Things J..

[15] El-Sheimy N. (2012). Inertial Techniques and Application, ENGO 623-Course Notes.

[16] Yu C., Lan H., Liu Z., El-Sheimy N., Yu F., Sun J., Liu J., Fan S., Wang F. (2016). Indoor Map Aiding/Map Matching Smartphone Navigation Using Auxiliary Particle Filter. China Satellite Navigation Conference (CSNC) 2016 Proceedings, Changsha, China, 18–20 May 2016.

[17] Lan H., Yu C., Li Y., Zhuang Y., El-Sheimy N. An efficient method for evaluating the performance of integrated multiple pedestrian navigation systems. Proceedings of the 2015 International Conference on Indoor Positioning and Indoor Navigation (IPIN).

[18] Niu X., Li Y., Zhang Q., Cheng Y., Shi C. (2012). Observability analysis of non-holonomic constraints for land-vehicle navigation systems. J. Glob. Position. Syst..

[19] Bahl V., Padmanabhan V. RADAR: An in-building RF-based user location and tracking system. Proceedings of the INFOCOM 2000: 19th Annual Joint Conference of the IEEE Computer and Communications Societies.

[20] Stella M., Russo M., Begušić D. (2014). Fingerprinting based localization in heterogeneous wireless networks. Expert Syst. Appl..

[21] Lan H., Yu C., El-Sheimy N., Sun J., Liu J., Fan S., Lu X. (2015). An integrated PDR/GNSS pedestrian navigation system. China Satellite Navigation Conference (CSNC) 2015 Proceedings, Xian, China, 13–15 May 2015.

[22] Gustafsson F. (2010). Particle filter theory and practice with positioning applications. IEEE Aerosp. Electron. Syst. Mag..

[23] Haykin S.S. (2001). Kalman Filtering and Neural Networks.

[24] Yu C., Lan H., Zhou Q., El-Sheimy N. PF for Indoor Map-Aided Navigation: Trade Off between Estimation Accuracy and Computational Speed. Proceedings of the 2016 International Technical Meeting of the Institute of Navigation.

[25] Arulampalam M.S., Maskell S., Gordon N., Clapp T. (2002). A tutorial on particle filters for online nonlinear/non-Gaussian Bayesian tracking. IEEE Trans. Signal Process..

[26] Pitt M.K., Shephard N. (1999). Filtering via simulation: Auxiliary particle filters. J. Am. Stat. Assoc..

[27] Renaudin V., Combettes C. (2014). Magnetic, Acceleration Fields and Gyroscope Quaternion (MAGYQ)-Based Attitude Estimation with Smartphone Sensors for Indoor Pedestrian Navigation. Sensors.

[28] Renaudin V., Susi M., Lachapelle G. (2012). Step Length Estimation Using Handheld Inertial Sensors. Sensors.

[29] Cho S.Y., Park C.G. (2006). MEMS based pedestrian navigation system. J. Navig..

[30] Grisetti G., Stachniss C., Burgard W. (2007). Improved Techniques for Grid Mapping with Rao-Blackwellized Particle Filters. IEEE Trans. Robot..

[31] Cossaboom M., Georgy J., Karamat T., Noureldin A. (2012). Augmented Kalman Filter and Map Matching for 3D RISS/GPS Integration for Land Vehicles. Int. J. Navig. Obs..

[32] Rothman Y., Klein I., Filin S. (2014). Analytical observability analysis of INS with vehicle constraints. Navigation.

[33] Zhuang Y., Lan H., Li Y., El-Sheimy N. (2015). PDR/INS/WiFi Integration Based on Handheld Devices for Indoor Pedestrian Navigation. Micromachines.

[34] Tang J., Chen Y., Chen L., Liu J., Hyyppä J., Kukko A., Kaartinen H., Hyyppä H., Chen R. (2015). Fast Fingerprint Database Maintenance for Indoor Positioning Based on UGV SLAM. Sensors.

[35] He X., Aloi D.N., Li J. (2015). Probabilistic Multi-Sensor Fusion Based Indoor Positioning System on a Mobile Device. Sensors.

[36] Groves P.D. (2013). Principles of GNSS, Inertial, and Multisensor Integrated Navigation Systems.

[37] Hong F., Zhang Y., Zhang Z., Wei M., Feng Y., Guo Z. WaP: Indoor localization and tracking using WiFi-Assisted Particle filter. Proceedings of the IEEE 39th Conference on Local Computer Networks (LCN).

